# Interaction of Chondroitin and Hyaluronan Glycosaminoglycans with Surfaces of Carboxylated Carbon Nanotubes Studied Using Molecular Dynamics Simulations

**DOI:** 10.3390/molecules28020826

**Published:** 2023-01-13

**Authors:** Tomasz Panczyk, Wojciech Plazinski, François-Yves Dupradeau, Agnieszka Brzyska, Pawel Wolski

**Affiliations:** 1 Jerzy Haber Institute of Catalysis and Surface Chemistry, Polish Academy of Sciences, Niezapominajek 8, 30239 Cracow, Poland; 2 Department of Biopharmacy, Medical University of Lublin, Chodzki 4a, 20093 Lublin, Poland; 3 AGIR, UFR de Pharmacie, Université de Picardie Jules Verne, Rue des Louvels, CEDEX 1, 80037 Amiens, France

**Keywords:** carbon nanotube, hyaluronan, chondroitin, adsorption, conformation, coating, molecular dynamics

## Abstract

Interaction of β-D-glucopyranuronic acid (GlcA), N-acetyl-β-D-glucosamine (GlcNAc), N-acetyl-β-D-galactosamine (GalNAc) and two natural decameric glycosaminoglycans, hyaluronic acid (HA) and Chondroitin (Ch) with carboxylated carbon nanotubes, were studied using molecular dynamics simulations in a condensed phase. The force field used for carbohydrates was the GLYCAM-06j version, while functionalized carbon nanotubes (fCNT) were described using version two of the general amber force field. We found a series of significant differences in carbohydrate-fCNT adsorption strength depending on the monosaccharide molecule and protonation state of surface carboxyl groups. GlcNAc and GalNAc reveal a strong adsorption on fCNT with deprotonated carboxyl groups, and a slightly weaker adsorption on the fCNT with protonated carboxyl groups. On the contrary, GlcA weakly adsorbs on fCNT. The change in protonation state of surface carboxyl groups leads to the reversal orientation of GlcNAc and GalNAc in reference to the fCNT surface, while GlcA is not sensitive to that factor. Adsorption of decameric oligomers on the surface of fCNT weakens with the increasing number of monosaccharide units. Chondroitin adsorbs weaker than hyaluronic acid and incorporation of four Ch molecules leads to partial detachment of them from the fCNT surface. The glycan–fCNT interactions are strong enough to alter the conformation of carbohydrate backbone; the corresponding conformational changes act toward a more intensive contact of glycan with the fCNT surface. Structural and energetic features of the adsorption process suggest the CH-π interaction-driven mechanism.

## 1. Introduction

The hydrophobic nature of carbon nanotube (CNT) surfaces limits their direct application in biomedical systems [1]. Therefore, a variety of surface modification methods have been applied in order to reduce their toxicity and to improve their solubility in aqueous media [2,3,4,5]. Particularly, the adsorption of hydrophilic and biocompatible moieties is a method of choice due to its simplicity and the lack of deterioration of the electronic and mechanical properties of CNT [6,7,8,9]. Among many others, saccharide molecules were studied as CNT surface modifying agents [10,11].

We have recently found that the adsorption strength of two monosaccharides β-D-glucopyranuronic acid (GlcA) and *N*-acetyl-β-D-glucosamine (GlcNAc) on CNT significantly differ. Among monosaccharides, only GlcNAc revealed the formation of stable surface layers. The adsorption strength of disaccharides, composed of GlcA and GlcNAc, and hyaluronan (HA) was found to be of a significant magnitude. However, those studies were focused on bare unfunctionalized CNT [11] which are rather idealized architectures. Indeed, commercially available CNT usually contain significant amounts (a few percent) of surface functional groups/defects.

The current investigations concern the interactions of carboxyl functionalized CNT (fCNT) with decameric HA, unfunctionalized glycosaminoglycan chondroitin (Ch, i.e., the structurally closest analog of HA) and monosaccharide building units: GlcNAc and GalNAc (*N*-acetyl-β-D-galactosamine). When neglecting both the possible sulfation sites along the oligosaccharide chain and the distinct mechanisms of interactions with proteins (non-covalent vs. non-covalent and covalent binding), the main difference between HA and Ch consists in the inversion of one chiral center within constituting disaccharide building blocks. This is equivalent to the exchange of GlcNAc (present in HA) into GalNAc (present in Ch). From a structural point of view, the position of the hydroxyl group at the C_4_ carbon atom (numbering in accordance with IUPAC-recommended nomenclature) in every second residue is altered from the equatorial (HA) to axial one (Ch). In view of our recent results [11], the equatorial position of ring substituents including inter-glycosidic oxygen atoms seems to play a key role in defining the strength of HA-CNT interactions. Such an observation is rather an exception than a rule, when considering the naturally occurring glycans, and is found only for saccharides composed exclusively of β-linked glucopyranose units. Therefore, apart from extending the range of studied compounds and accounting for the possible functionalization of CNT, our present investigation aims to check if extending the bioavailability of fCNT can be effectively achieved by using a broader set of natural glycosaminoglycans, with alternative positions of substituents.

Classical force fields and their empirical functional forms have a series of limitations, that may hinder elucidating the exact nature of the interactions responsible for the glycan-CNT binding. Thus, our previous studies relied on classical simulations, but are also supported using quantum-mechanical calculations [11] demonstrating that the adopted methodology successfully explains the driving forces responsible for the adsorption of HA onto bare unfunctionalized CNT. More precisely, we stated that the attractive, CH-π interactions between CNT and axial aliphatic hydrogen atoms of HA are the driving force for the binding process. These results are supported by the energetically favorable reduction of the non-polar surface of CNT and hydrophobic patches within carbohydrate rings. This outcome is analogous to that observed in the mechanism occurring in the binding of neutral carbohydrates with proteins [12,13]. The conformational properties of the currently studied glycans [14] strongly suggest that in these cases the dominating mechanism of adsorption may be the same. However, a series of alterations is expected to occur, resulting, for e.g., from structural differences between HA and Ch and the functionalization of CNT.

## 2. Results and Discussion

### 2.1. Simulation Setup

The current study is mainly focused on the effects of fCNT on the adsorption of saccharides on their external surfaces. Therefore, the starting point in modeling these compounds is the construction of the fCNT model. CNT are often purified using oxidative agents [2,4,15].

**Figure 1:**
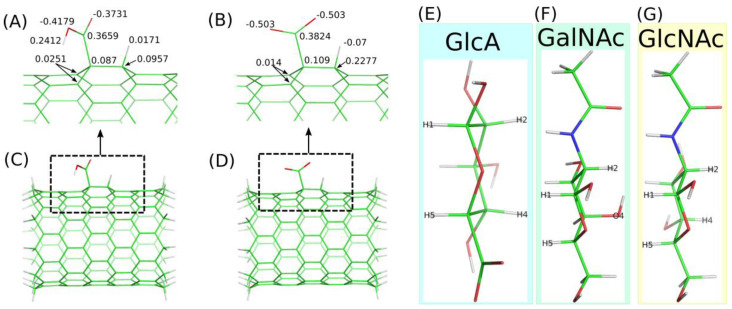
(**A**,**B**) show the schematic representation of fCNT with a carboxylic acid or a carboxylate (COOH and COO^⊖^), respectively. The topology of bonds formed after grafting the functional groups into the CNT structure, and atomic charges on atoms involved in the CNT functionalization are showed. (**C**,**D**) show small (10,0) CNT clusters with COOH and COO^⊖^, respectively, used in quantum mechanics computation and RESP atomic charges derivation using B3LYP/6-31G(d) implemented in RED Server Development. Green sticks represent carbon atoms, red ones oxygen atoms, and grey ones hydrogen atoms. (**E**–**G**) show stick representations of monosaccharides involved in this study together with labels on the atoms, which help to define spatial orientation of a given molecule in reference to the fCNT surface (atom numbering remains in accordance with IUPAC recommendations).

Thus, a common functional group present on the CNT surface is the carboxyl group. In aqueous dispersion, fCNT carboxyl groups partly dissociate depending on the pH leading to the carboxylic acid or carboxylate function. Thus, in order to fully understand the possible role of saccharides as agents tweaking the biocompatibility and hydrophilicity of fCNT surfaces, both the carboxylic acid and carboxylate forms of the carboxyl group have to be studied. In order to generate fCNT we assumed that the presence of carboxyl groups (either protonated or deprotonated) leads to the rehybridization (from sp^2^ to sp^3^) of the carbon atom to which the carboxyl group is bound. Also, the neighboring carbon atom needs similar rehybridization in order to keep the valence conserved. This is schematically shown in Figure 1 together with the partial charges on atoms directly or indirectly involved in the functionalization scheme.

The detailed description of the empirical force field generation for fCNT is available in the Methods section. Here we need to mention several assumptions made during model building. One such assumption is that the presence of the functional groups leads to atomic charge values not equal to zero for these groups and for the neighboring CNT carbon atoms. The values of those charges are shown in Figure 1A,B. These partial charges were derived using the RESP-X1 scheme for fitting quantum chemically determined electrostatic potentials into point charges [16,17]. Particular attention was paid to chemical and charge equivalencing for the fCNT atoms to derive equivalent charge values for chemically equivalent atoms. This was achieved by using the RED Server Development service which implements the methodology described by Vanquelef et al. [18]. 

Small clusters of (10,0) CNT were used in these cases as models of carboxylated fCNT. These models are shown in Figure 1C,D and contain 164 and 165 atoms for CNT-COO^⊖^ and CNT-COOH, respectively. Nevertheless, the density-functional theory calculation for optimizing geometries and computing molecular electrostatic potentials was used; the combination of functional and basis set was B3LYP/6-31G(d). 

The derived charge values (Figure 1A,B) were used during the grafting of each carboxylic acid/carboxylate group to the CNT structure. The total number of these groups of atoms were assumed to represent 2% of the CNT carbon atoms. This is a rather common value reported in commercially available samples. We used (17,0) CNT with a length of 5.9 nm: the number of functional groups was 20 per CNT. The incorporation of these groups of atoms into the large CNT molecule needed charge adjustment for other atoms. This was achieved by dividing the total residual charge determined from each group by the number of carbon atoms in the CNT. Thus, each atom in the fCNT bears a charge value close to zero in order to compensate the charges computed for the functional groups.

The fCNT was treated as an infinite molecule by applying periodic replicas along its axis for bonds, angles, dihedrals and all other factors. The box size was adjusted to the length of the main (17,0) fCNT and the number of carbon atoms in that nanotube was 952. The fCNT was initially put in the middle of a simulation box, and saccharide molecules were placed in a way that prevents overlaps with fCNT or with other molecules. In this work we investigated the interaction of fCNT with monosacchrides such as β-D-glucopyranuronic acid (GlcA), N-acetyl-β-D-glucosamine (GlcNAc) and N-acetyl-β-D-galactosamine (GalNAc). Additionally, we also studied the interaction of two oligosaccharides: hyaluronic acid (HA) and chondroitin (Ch), both composed of monosaccharide decamers with a GlcA-β-1,3-GlcNAc- and GlcA-β-1,3-GalNAc- repetitive pattern, respectively. Figure 1E–G show stick representation of monosaccharides utilized in the calculations and they also show atom name labels on hydrogen and oxygen atoms, which are used to analyze the carbohydrate orientation in reference to the fCNT surface (according to IUPAC nomenclature). 

The combination of carboxylic acid and carboxylate fCNT (CNT-COOH and CNT-COO^⊖^) and the considered monosaccharides and oligosaccharides lead to ten different systems. Additionally, we also studied four other molecular systems with four oligosaccharides interacting with a single fCNT and a single Ch chain system alone in water in order to estimate the influence of fCNT on the conformational properties of the glycan chain. In this context, the analogous data for the HA chain were taken from our previous paper [11]. 

### 2.2. Adsorption of Monosaccharides on fCNT 

**Figure 2:**
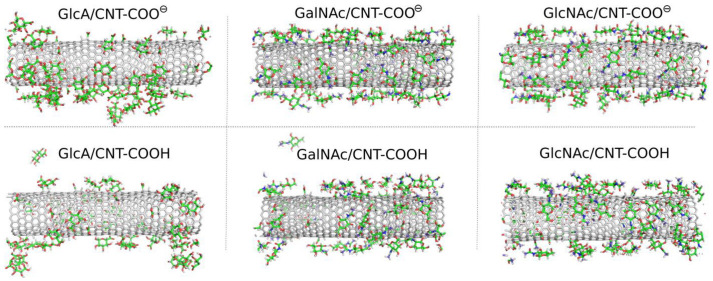
MD simulation snapshots taken at the last simulation frames, i.e., after 600 ns for the fCNT systems with monosaccharides. The temperature and pressure were 310 K and 1 bar, respectively.

The study of the interaction of monosaccharides GlcA, GalNAc and GlcNAc with CNT-COOH and CNT-COO^⊖^ using molecular dynamics (MD) simulations in a condensed phase led to significant qualitative differences between the considered cases. Visual inspection of the MD snapshots is useful for drawing the first qualitative conclusions about the properties of systems under investigation. Thus, Figure 2 shows the MD snapshots for monosaccharide adsorption taken at the last simulation frames, i.e., after 600 ns of the production runs. 

As described in Figure 2 the monosaccharides attach to the surfaces of the fCNT, but in some cases individual molecules were found also in the bulk water. In the GlcA/CNT-COOH and GalNAc/CNT-COOH molecular systems the adsorption is considered as unstable. This is because in a possible experimental situation, the re-adsorption of temporarily desorbed molecules is unlikely to be observed. This is due to the much larger volume of the aqueous phase in a possible experimental system. During MD simulations the re-adsorption occurs relatively quickly due to the rather small distance necessary to be passed by the desorbed molecule and to interact with a fCNT image in the periodic conditions. 

Thus, individual events of spontaneous detachments seen in MD simulations mean that in experimental conditions the adsorption/desorption events will be occurring simultaneously (reversible adsorption). The effective surface coverage will be strongly dependent on the bulk concentration of monosaccharides and temperature. 

**Figure 3:**
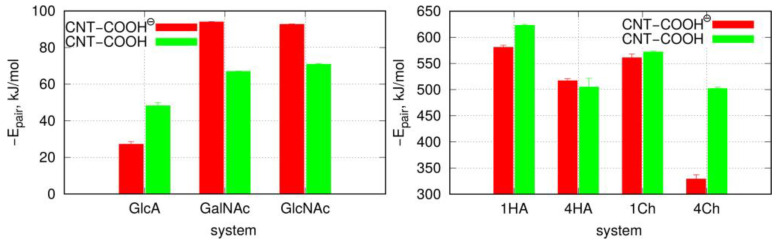
Total mean-pair-interaction energies from Table 1 plotted as bar charts. The left panel stands for the GlcA, GalNAc and GlcNAc monosaccharides, while the right panel stands for HA and Ch oligosaccharides with a single (1HA and 1Ch) and four (4HA and 4Ch) carbohydrates on the fCNT surface.

A more quantitative analysis of the adsorption of monosaccharides on the fCNT surface was based on the determination of pair-interaction energies between fCNT and adsorbed molecules and also depends on the determination of the radial density profiles (RDF) of adsorbed molecules in reference to the fCNT axis. These results are collected in Table 1 and Figure 2 and Figure 3. Thus, Table 1 shows the determined mean-pair-interaction energies for the different studied molecular systems. These energies are split into the Lennard–Jones and electrostatic contributions. Figure 2 in turn shows the total pair interaction energies as bar charts for the molecular systems. 

The qualitative conclusion drawn from the MD simulation snapshots are supported by the analysis of pair-interaction energies between adsorbed carbohydrates and fCNT. Thus, the strongest interaction is observed for GalNAc and GlcNAc and deprotonated fCNT. This is quite surprising since these molecules are neutral. Moreover, a significant contribution from the electrostatic interactions to the total energy (−26 kJ mol^−1^ as seen in Table 1) was not expected. The CNT-COOH protonated molecular systems actually reveal a similar range of dispersion interactions, with a significantly smaller contribution for the Coulomb energies. Intuitively, the electrostatic energy values for negatively charged fCNT with neutral monosaccharides are not expected to be large. However, the observations are clear: the contribution of the electrostatic interactions for the studied monosaccharides is significant and leads to a qualitative change in properties for the analyzed systems.

The properties of negatively charged GlcA with fCNT is more intuitive: the neutral CNT-COOH interacts more strongly with GlcA molecules than the negatively charged CNT-COO^⊖^. However, the difference is not that large when looking at the total energies. Nevertheless, these two molecular systems are definitely the weakest in terms of the adsorption strengths when compared to GlcNAc and GalNAc. 

**Figure 4:**
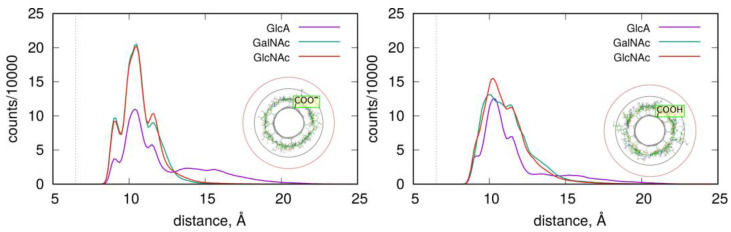
RDF of the GlcA, GalNAc and GlcNAc monosaccharides adsorbed on the fCNT surfaces as a function of the distance from the fCNT axis. The dashed vertical lines show the position of carbon atoms within the nanotube. The concentric black and red circles show the 15 Å and 20 Å distances, respectively, from the fCNT axis using the last MD simulation frames from GlcNAc systems as background images. The number of counts reports the number of times a given distance was detected during the production run.

The structures and stabilities of the adsorbed layers are more quantitatively described using the RDF functions of the monosaccharides in reference to the fCNT axis. Figure 4 shows the corresponding data for the studied systems.

As described in Figure 4, the adsorbed layers are the most compact in the case of GalNAc and GlcNAc adsorbed at the CNT-COO^⊖^ surface. This is in agreement with the pair-interaction energies reported in Figure 3 and Table 1. In these cases, all monosaccharide atoms fit within a distance smaller than 15 Å from the nanotube axis. As observed in insets in Figure 4, this distance corresponds to the monolayer coverage of the nanotube surface. However, in all cases, the densities decay is almost at zero within the distance 20 Å from the fCNT axis. The non-zero density within the 15 Å–20 Å means that the adsorbed layers are more diffuse or that transient detachments of the adsorbed molecules occur. This happens to the GlcA for both fCNT types as well as GlcNAc and GalNAc in the case of protonated fCNT.

The shapes of the density profiles differ significantly depending on the protonation state of the fCNT surface in the cases of GlcNAc and GalNAc. It seems that fCNT-carboxylate groups lead to the formation of stiffer monosaccharide–fCNT complexes and well-defined peaks on the distributions. In the cases of fCNT carboxylic acid groups, the adsorbed layers are more loosely defined and the density profiles are more diffuse. 

Interestingly, both GlcNAc and GalNAc monosaccharides display nearly the same density profiles and interaction energies, independently of the fCNT type. This suggests that the glucose to galactose epimerization at the C4 atom is not of primary relevance for the structural features of adsorbed layer, nor for the energetic properties of adsorption.

**Figure 5:**
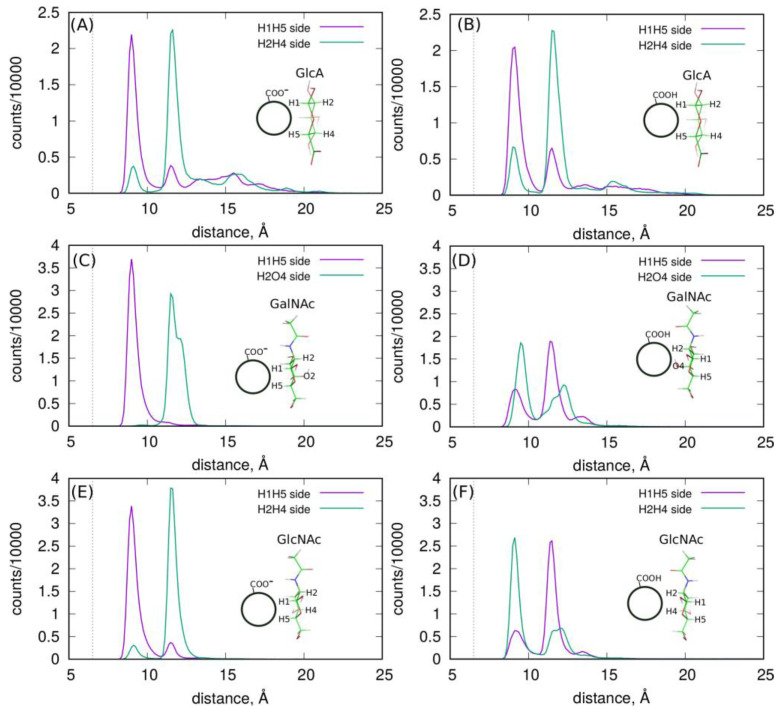
The density profiles of monosaccharide H1–H5 and H2–H4(O4) atoms in each molecular system. Parts (**A**,**B**) are for GlcA, parts (**C**,**D**) are for GalNAc and parts (**E**,**F**) are for GlcNAc. The insets in each graph show the observed preferred orientation of the monosaccharide in reference to the fCNT surface. These orientations correspond to the pair of atoms, for which the first peak appears on the density distribution.

More detailed information about the structures of adsorbed layers comes from the analysis of the orientations of the GlcA, GalNAc and GlcNAc monosaccharide at/on the fCNT surfaces. These orientations can be defined by the density profiles of some selected atoms, which are shown in Figure 1. Each monosaccharide has a distinct “side” defined by the arrangement of H1–H5 and H2–H4 (or O4) atoms, as described in Figure 1E–G. Thus, determination of the density profiles of the H1–H5 and H2–H4(O4) pairs gives direct information about the preferred orientation of a monosaccharide on the fCNT surface. The obtained results are shown in Figure 5.

As shown, each monosaccharide has a preferred orientation towards the fCNT surface. A similar observation was reported in the analysis of HA oligosaccharide adsorption on the surfaces of bare unfunctionalized CNT in a previous work [11]. It was found that GlcA and GlcNAc prefer to be adsorbed via the H1-H5 and H2-H4 side, respectively. The current study provides even more striking results since we conclude that the protonation state of the fCNT affects the preferred orientation of GlcNAc and GalNAc. In this new work, GlcA still reveals the preferred H1-H5 orientation no matter what the protonation state of the fCNT is. Moreover, GlcNAc and GalNAc display a preferred orientation with their H1-H5 side towards the CNT-COO^⊖^ surface, and on the other side towards the CNT-COOH surface. This means that these changes can be externally controlled by the changes in the pH of the aqueous solution. Thus, this new property of the monosaccharides/fCNT systems can be utilized in practical realizations/experimental trials of molecular nanodevices and sensors. 

Finally, the clear preferences of monosaccharides to interact with CNT via the face-to-face contact of their aliphatic patches with the CNT surface is a clear indicator of CH-π-attractive interactions. Such interactions were unambiguously demonstrated in our previous study [11] as being responsible for the adsorption of HA onto the surface of bare unfunctionalized CNT. Similar interactions are responsible for the binding of carbohydrates to proteins [13,19] where the role of π donor is played by aromatic amino acids.

### 2.3. Adsorption of Oligosaccharides on fCNTs

**Figure 6:**
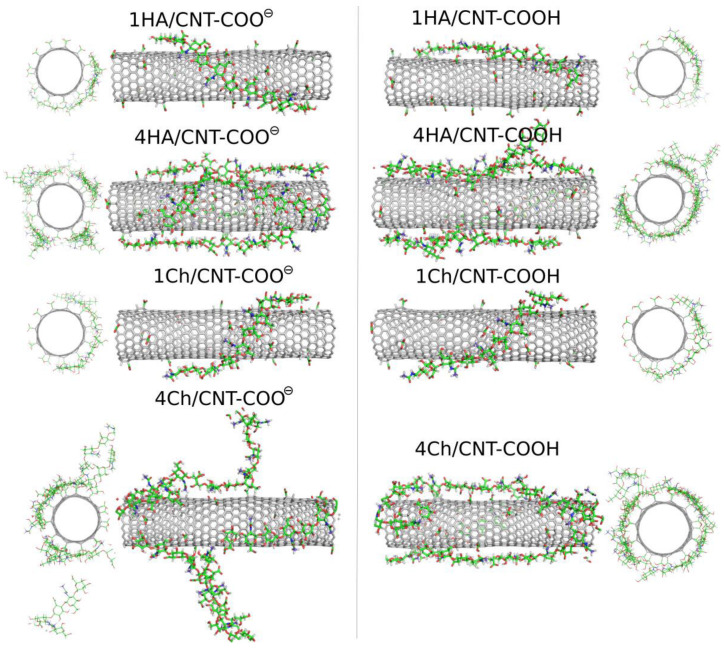
MD simulation snapshots for molecular systems involving the HA and Ch oligosaccharides: HA (one or four HA molecules) and Ch (one or four Ch molecules) adsorbed on the CNT-COOH and CNT-COO^⊖^ surfaces.

Oligosaccharides can potentially be more useful as CNT surface-modifying agents due to their larger molar mass and their stronger binding to the surface. In this study, we analyzed the adsorption behavior of decameric HA and decameric Ch chains. We start the analysis of the results from the visual inspection of the simulation boxes at the final step, i.e., after 600 ns of productive MD simulations. Figure 6 shows these snapshots for the studied systems involving oligosaccharides.

The qualitative behavior of HA and Ch oligosaccharides on fCNT surfaces can be summarized as follows. The single decamers tightly wrap the fCNT no matter what the considered oligosaccharides and fCNT are. However, at a higher concentration of oligosaccharides represented by four decamers with a fCNT, destabilization of the adsorption is observed. Particularly, in the four Ch/CNT-COO^⊖^ system, Ch oligosaccharides show a predilection for partial detachments from the fCNT surface and tend to stand perpendicularly to the fCNT axis. Such behavior is not observed in the case of HA or Ch adsorbed on CNT-COOH and is therefore not an effect of the crowded conditions. Elsewhere, the free energy component coming from the interaction between Ch molecules and fCNT is comparable to that for the interaction with water. As seen in Table 1 and Figure 3, increasing the number of the adsorbed molecules reduces the effective pair interaction per single molecule. Thus, the balance between free energy components from fCNT and water becomes shifted towards water as the number of Ch molecules increases. This leads to the unstable adsorption of Ch on fCNT with carboxylate groups. We can further conclude that changing the pH so that the surface carboxyl groups switch from the protonated to the deprotonated state (or vice versa) should lead to the reversible adsorption and desorption of Ch molecules on fCNT. This effect is not observed for HA molecules, at least in the considered conditions, i.e., up to four HA molecules on the (17,0) 5.9 nm long fCNT.

**Figure 7:**
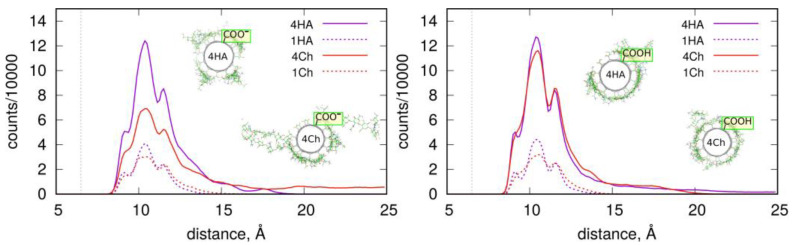
RDF of the HA (one and four HA) and Ch (one and four Ch) oligosaccharides adsorbed on the fCNT surfaces as a function of the distance from the fCNT axis. The dashed lines show the profiles of single molecule adsorption, while the solid lines are for the adsorption of four molecules.

These conclusions are also supported by the RDF, which contain information from the whole-productive MD simulations (as for energy values in Table 1). Figure 7 shows such profiles, which are analogous to those in Figure 4. The density of four Ch oligosaccharides at the CNT-COO^⊖^ surface do not decay to zero. This means that perpendicularly standing Ch oligosaccharides are observed during the whole-productive MD simulations. In the case of CNT-COOH, the adsorbed layer of the four Ch decamers terminates at 20 Å, which demonstrate the presence of a single layer and a stable adsorption. Interestingly, the four HA/CNT-COOH system reveals a long tail on the RDF indicating similar behavior to the Ch/CNT-COOH system, but with a much weaker intensity.

Single-molecule adsorption is in both cases strong and limited strictly to the single-layer occupation without any transient displacement from the fCNT surface. This conclusion comes from either the pair-interaction energies shown in Table 1 and Figure 3 or from the fact that RDF never exceed 15 Å, as seen in Figure 7. Increasing the concentration of oligosaccharides leads to the reduction of the pair-interaction energy with fCNT, and to significant displacements of oligosaccharides from the fCNT surfaces.

### 2.4. Hydrogen Bonds (HB)

Carboxyl groups located on the fCNT surface can potentially take part in the formation of HB with carbohydrate molecules. The existence or non-existence of such bonds allows us to better understand the adsorption mechanism. Thus, depending on the protonation state of the carboxyl group, the later can act as an acceptor only (carboxylate group) or as acceptor and donor (carboxylic acid group). The results of the analysis of the number of HB are collected in Table 2.

The formal definition of the hydrogen bond usually relies on geometric factors such as distance and angle, though in the literature definitions also based on energetic factors were applied [20]. Here, for simplicity, we assume that a strict HB exists when the distance between the acceptor and donor is smaller than 3 Å and the angle acceptor-donor-H is less than 20 degrees. We do not count so-called weak HB which corresponds to a larger distance threshold 3.5 Å. This is because we are mainly interested in highly specific and strong interactions between the considered compounds.

HB are actually absent in the case of CNT-COOH. Either the acceptor or donor function of the carboxylic acid groups do not form any HB, though in the cases of GalNAc and GlcNAc one HB of the formally possible forty bonds was detected. The donor function of the carboxylic acid groups is absolutely negligible in all the cases. In turn, the carboxylate groups take part in the formation of a significant number of HB with GalNAc and GlcNAc. Thus, the observed nine HB means that the presence of HB represents a key contribution to the structural stability of the adsorbed layer of the GalNAc/CNT-COO^⊖^ and GlcNAc/CNT-COO^⊖^ surfaces. Other molecular systems reveal smaller number of HB, and in particular the low values observed for the 1HA and 1Ch systems are mainly related to the geometrical difficulty of approaching oxygen atoms distributed randomly on the surface by the rather stiff oligosaccharides chains. Finally, a visual inspection of the orientations of monosaccharides molecules taking part in HB does not imply that the HB are responsible for their specific alignment on the surface, as discussed in reference to Figure 5.

### 2.5. Influence of Glycan Adsorption on Its Conformation

As the CNT molecule displays a significant degree of rigidity, we did not analyze their potential structural alterations induced by the presence of adsorbed saccharides. On the contrary, carbohydrate molecules are conformationally heterogeneous and deserving of a systematic analysis. We studied how the conformation of HA and Ch decamers is affected by CNT, when the complex is formed. Thus, free energy maps were recovered from MD trajectories by using the main conformational descriptors, illustrating the three-dimensional shape of the glycosaminoglycan backbone, i.e., the ϕ and ψ glycosidic linkage torsion angles as defined in the Methods section.

**Figure 8:**
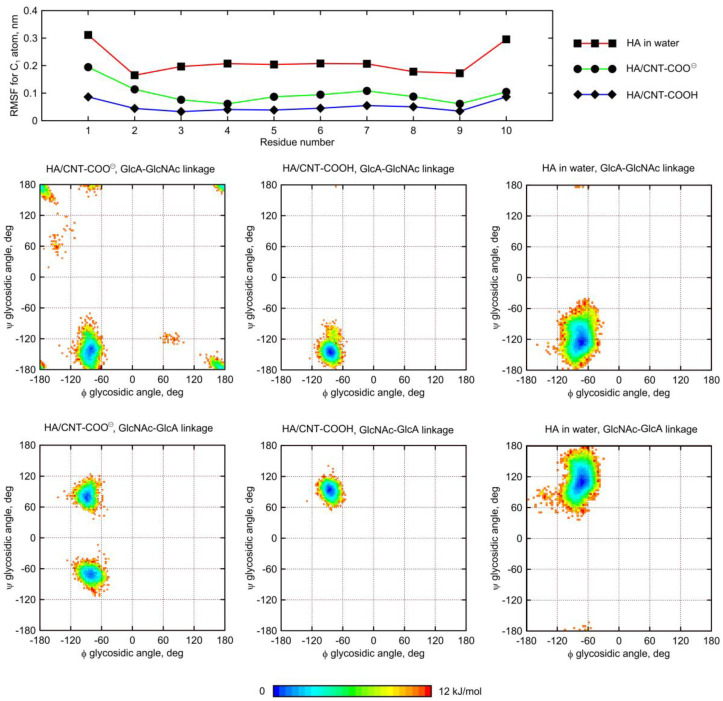
(**top**) Root-mean-square-fluctuation (RMSF) parameter values calculated for anomeric carbon atoms in the HA decamer. (**bottom**) Free energy maps calculated from unbiased MD trajectories in the glycosidic dihedral angle ϕ, ψ coordinates for the HA molecule. Free energy values were determined by using the Boltzmann inversion method. The calculations included both types of glycosidic linkages present in the oligosaccharide decamer and the two possible states of the glycan molecules, i.e., either free in water (HA in water in the legend) or adsorbed on the fCNT (HA/CNT-COOH and HA/CNT-COO^⊖^ in the legend) surface. The energy scale is in [kJ/mol].

Figure 8 shows the free energy maps generated for the HA decamer in the glycosidic dihedral angle ϕ, ψ coordinates. The most notable observed effect of HA adsorption on fCNT is the reduction of its conformational space in comparison to that of the free glycan molecule. This is due to the influence of external forces originating from the presence of CNT and the limited mobility of the adsorbed oligosaccharide. When adsorbed onto CNT-COO^⊖^, the HA conformational space shows significant alterations. Namely, the secondary glycosidic-linkage-type of geometry shapes appears. Based on the production MD simulations, the presence of this alternative conformation is associated with the existence of conformationally locked ‘kinks’ in the adsorbed HA decamer, while the rate of conformational exchanges involving reorientation of linkages is too slow to fully recover the conformational, dynamic equilibria.

The discussed changes in backbone geometry take place in the β(1–4) and β(1–3) glycosidic linkage present in HA, but the first one seems to be affected to a larger extent in comparison to the second one, as the populations of *syn*- and *anti*- conformers [21] are nearly equal.

Our previous work [11], which focused on the HA/unfunctionalized–CNT interaction resulted in similar findings, showing that the adsorption of HA is associated with conformational alterations of the HA backbone. This occurs via the rotation of the ψ dihedral angle. In spite of the qualitatively distinct behaviors observed in the cases of CNT-COO^⊖^ and CNT-COOH (conformational alteration of backbone for the ψ dihedral angle vs. no alteration, respectively), we refrain from drawing any ultimate conclusion about the influence of CNT on the dynamics and the conformational feature of adsorbed glycan decamers. This is because the production MD simulation time is not sufficiently long enough to observe multiple binding/unbinding events and, consequently, may not correctly render the dynamics of the ϕ and ψ dihedral angles.

Nevertheless, it is clear that the fCNT–HA interactions are strong enough to perturb the intrinsic dynamic equilibrium related to the conformation of the glycosidic linkage within the HA oligosaccharide.

**Figure 9:**
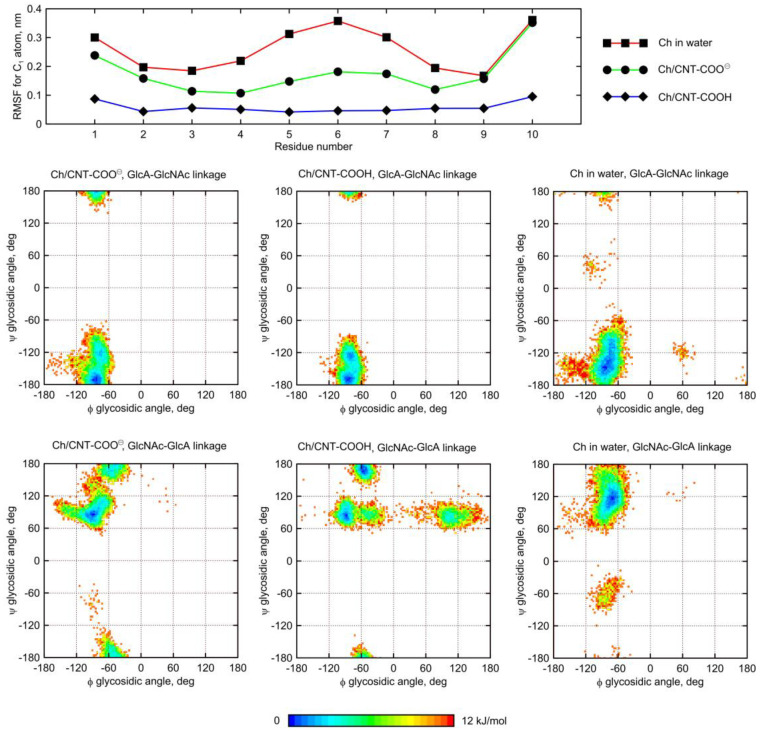
(**top**) Root-mean-square-fluctuation (RMSF) parameter values calculated for anomeric carbon atoms in the Ch decamer. (**bottom**) Free energy maps calculated from unbiased MD trajectories in the coordinates the glycosidic dihedral angle ϕ, ψ coordinates for the Ch molecule (details in Figure 8).

A similar behavior was observed for the Ch oligosaccharide (Figure 9). However, the largest deformation of the glycosidic linkage is observed as a result of adsorption on CNT-COOH, whereas interactions of Ch with CNT-COO^⊖^ display less significant differences. Interestingly, instead of expected *anti-*ψ conformations, the glycosidic bonds of the adsorbed Ch chain now explore the quasi *trans-*ψ conformation. This unusual behavior is observed in the case of both β(1–4) and β(1–3) linkages, and for CNT-COO^⊖^ and CNT-COOH. Moreover, the deformation along the ϕ dihedral angle is observed, but only in the case of CNT-COO^⊖^ and the β(1–4) linkage. Such changes in the ϕ value act against the exo-anomeric effect [22] and, again, speaks for strong, attractive forces acting between glycans and fCNT. Apart from that, the expected reduction in the ϕ, ψ conformational space upon adsorption onto CNT is observed as well.

The dynamic geometry of glycan oligosaccharides affected by the CNT presence is also illustrated by the changes in root-mean-square-fluctuation (RMSF) parameter values (Figure 8 and Figure 9). Large decreases of the RMSF values calculated for the anomeric carbon atoms and adsorbed glycan chains speak for reduced mobilities of saccharides adsorbed on CNT and agree with both the monitored alterations of conformational properties and the expectations. The reduction of RMSF values upon adsorption on CNT-COO^⊖^ is similar to that observed for HA-unfunctionalized–CNT interactions [11] and equal to ca. 50%. However much larger reduction (even up to 80%) is observed in the case of both HA and Ch interacting with CNT-COOH. This is in line with pair-interaction energies determined for oligosaccharides (Figure 3) and visual inspection of the behavior of oligosaccharides adsorbed on fCNT.

Finally, in the case of both HA and Ch, we observed several events of pyranose ring distortion, changing the ring geometry from ^4^C_1_ to boat and skew-boat shapes. This effect concerns mainly GlcA units, in which the carboxylate group is forced to adopt the axial or quasi-axial conformation, and is located further from the CNT surface. Again, this observation can be interpreted in terms of strong saccharide-CNT affinity, which results in forces that emerge on the ring substituent and shift the dynamic equilibria of the ring conformation [23].

Summarizing, the conformation of glycosidic linkages in the adsorbed saccharides may undergo significant alterations in order to facilitate the favorable CNT–glycan interactions and increase the area of the glycan-fCNT contact. The observed kinks in glycosidic linkages as well as the presence of alternative ring shapes are conformationally locked and are indicators of strong, attractive interactions between rings of adsorbed saccharides and fCNT.

## 3. Methods

### 3.1. Carbon Nanotubes

The atomic structure of the studied fCNT were generated using self-designed scripts. We started with (17,0) unfunctionalized CNT. As mentioned in Section 2.1, CNT has been subjected to functionalization using the carboxylate and carboxlic acid groups. These groups were grafted to randomly selected carbon atoms on the surface of the nanotube. The number of functional groups was set to twenty, which corresponds to 2% of the total number of CNT atoms. The assumed length of the CNT was 5.9 nm, and it was treated as periodic molecule in the direction of the CNT axis. The topology of the force field for the functionalized CNT was generated using the AcPyPe script [24] which interfaces a suite of programs from the AmberTools package (https://ambermd.org, accessed on 1 June 2022) [25,26]. Because the fCNTs do not belong to any standard class of organic molecules, we utilized the general amber force field, gaff2 [27], with parameters set for the force field parameters values [16,17], as reported in Section 2.1. The described approach leads to the force field, which treats the fCNT as a large molecule with only pairwise-additive interactions. Indeed, we found that this simple description is accurate enough as the CNT wall structure was close to that generated via the many-body ai-REBO force field [28].

### 3.2. Carbohydrates

The MD simulations involved a set of monosaccharides and oligosaccharides representing two naturally-occurring glycosaminoglycans, i.e., HA and non-sulfated Ch. The selected monosaccharides are building blocks for these two glycans; GlcA residues are included in both HA and Ch, whereas GlcNAc and GalNAc belong to HA and Ch, respectively. Considered oligosaccharides consisted of decameric fragments of either HA or Ch chains. The reducing-ends in all carbohydrates were always left unfunctionalized, and the anomer type corresponded to β-glycosidic linkage in a glycan chain. The carboxylic acid group of the GlcA residue was deprotonated, according to its pK*a* value, and assumed physiological conditions of pH. The carbohydrate-dedicated GLYCAM-06j [29] force field parameters were generated using the Glycam Carbohydrate Builder online server (glycam.org). The GLYCAM parameters are capable of reproducing the CH-π interactions [12] and expected to play a significant role in the studied molecular systems. The conformation of glycosidic linkages in the glycan backbone was defined using the φ and ψ glycosidic torsional angles, in accordance with the IUPAC recommendations (φ = O_5_-C_1_-O_1_-C’*_n_*; ψ = C_1_-O_1_-C’*_n_*-C’*_n_*_-1_, where *n* = 4 or 3, depending on the linkage type).

### 3.3. Computational Methodology

MD simulations were carried out by using the Gromacs 2016.4 program [30] with the input parameters for fCNT and carbohydrates generated in the way described in Section 3.1 and Section 3.2. The simulation boxes were generated using self-designed script, which inserted the fCNT in the middle of the x, y box dimensions, and placed carbohydrate molecules around the fCNT at distances which prevent overlaps. Next the sodium and chloride ions were inserted at random positions in the box, and their amounts were calculated assuming an ionic strength of solution 0.15 mol L^−1^ and keeping the charge neutrality of the solution due to insertion of either negatively charged fCNT or GlcA molecules. The ions were described using parameter values from the amber force field [26], i.e., ε_LJ_(Na^+^) = 0.366 kJ/mol; σ_LJ_(Na^+^) = 0.244 nm; ε_LJ_(Cl^−^) = 0.149 kJ/mol; σ_LJ_(Cl^−^) = 0.448 nm. Finally, water TIP3P [31] molecules (ca. 4200–4500) were inserted in the box. The initial dimension of the box was 5.5 nm × 5.5 nm × 5.952 nm.

The simulations were performed at constant pressure 1bar, controlled using Parrinello-Rahman barostat [32], and constant temperature 310 K, controlled using the velocity rescaling Berendsen thermostat [33]. The electrostatic interactions were calculated using the particle mesh Ewald summation [34] with the cutoff 1.2 nm for both electrostatic and Lennard–Jones interactions. The integration timestep was 2 fs. The equilibration runs were carried out for 80 ns for each system, and the production runs corresponded to 600 ns of real time for each system. The snapshots of the trajectories and energies were conducted every 200 ps. So, each production run consisted of 3000 independent samples.

## 4. Summary and Conclusions

In this work, we have described the results of a MD study in the condensed phase concerning the interaction of HA, Ch and their constitutive building blocks GlcA, GlcNAc and GalNAc with carboxylated fCNT. The carbohydrate-fCNT complexes were analyzed mainly in the context of the production of biocompatible CNT using non-covalent surface modification agents via the adsorption of naturally occurring monosaccharides and oligosaccharides. Structural factors within the oligosaccharides upon adsorption on fCNT were discussed.

The conclusions concerning the potential protective role of saccharides are generally not straightforward. The behavior of GlcA was not surprising and similar to a previous study concerning the application of bare unfunctionalized CNT [11]. Thus, GlcA adsorption is unstable on the fCNT no matter what the protonation state of the carboxyl groups is. However, GlcA adsorbs with its H1-H5 side on the fCNT surfaces, and the adsorption is reversible at the considered conditions. The behavior of GalNAc and GlcNAc is almost identical and intriguing. Namely, both molecules adsorb strongly on CNT-COO^⊖^ and slightly weaker on CNT-COOH. But after switching the protonation state of the carboxyl group, the monosaccharide molecules reverse their orientation on the fCNT surface. The orientation of these molecules on the CNT-COO^⊖^ is clearly identified as H1–H5 side laying on the fCNT surface. Decreasing pH and switching into CNT-COOH leads to H2–H4(O4) side laying on the fCNT surface. This property is useful, when designing pH switchable nanodevices, for instance.

The glycan-fCNT contact patterns are compatible with those observed previously for interactions of HA, and its building blocks with unfunctionalized CNTs; they confirm the contribution of CH-π interactions as the main driving force for glycan-CNT adsorption. Adsorption of the HA and Ch oligosaccharides is also rather surprising since in both cases increasing the number of HA or Ch molecules leads to the weakening of their adsorption. Single oligosaccharide molecules adsorb rather strongly and irreversibly at the considered conditions. However, increasing the concentration leads to the destabilization of the adsorption, particularly in the Ch case, where almost total detachment of Ch molecules was observed.

The process of adsorption of glycan chains onto fCNT surfaces significantly influences the conformation of adsorbed molecules. A series of conformationally locked rearrangements are observed, such as the deformation of key glycosidic linkages within the glycan backbone and the distortion of the GlcA chair conformation. All these alterations act toward tighter glycan-fCNT contacts, and are the consequences of strong, attractive interactions between the two species.

To sum up, short oligosaccharides reveal rather limited potential as surface protective layers of commonly used carboxylated carbon nanotubes. On the other hand, selected monosaccharides reveal interesting behavior related to their specific orientation on the fCNTs surfaces and their reversal triggered by pH changes.

## Figures and Tables

**Table 1:** Mean-pair-interaction energies between the fCNT and the carbohydrate molecules. The energies are calculated per single carbohydrate molecule and expressed in kJ mol^−1^. The energies are split into the Lennard–Jones and the Coulomb contributions.

Molecular Systems	Lennard–Jones (kJ mol^−1^)	Coulomb (kJ mol^−1^)	Total (kJ mol^−1^)
GlcA/CNT-COO^⊖^	−40.1 ± 1.4	12.9 ± 1.4	−27.2 ± 1.4
GlcA/CNT-COOH	−49.6 ± 1.6	1.5 ± 0.4	−48.2 ± 1.6
GalNAc/CNT-COO^⊖^	−67.1 ± 0.2	−26.9 ± 0.2	−94.0 ± 0.2
GalNAc/CNT-COOH	−61.1 ± 0.1	−6.0 ± 0.1	−67.0 ± 0.1
GlcNAc/CNT-COO^⊖^	−67.2 ± 0.2	−25.6 ± 0.1	−92.7 ± 0.2
GlcNAc/CNT-COOH	−65.0 ± 0.4	−5.9 ± 0.1	−70.9 ± 0.4
4HA/CNT-COO^⊖^	−486 ± 4.0	−30.6 ± 0.1	−517 ± 4.0
1HA/CNT-COO^⊖^	−570 ± 1.5	−11.0 ± 3.5	−581 ± 3.8
4HA/CNT-COOH	−486 ± 16.5	−18.3 ± 1.8	−505 ± 16.6
1HA/CNT-COOH	−606 ± 1.0	−17.0 ± 1.6	−623 ± 1.9
4Ch/CNT-COO^⊖^	−299 ± 3.3	−30.0 ± 7.3	−329 ± 8.0
1Ch/CNT-COO^⊖^	−519 ± 5.2	−42.0 ± 4.8	−561 ± 7.1
4Ch/CNT-COOH	−479 ± 2.7	−24.0 ± 1.0	−502 ± 2.9
1Ch/CNT-COOH	−546 ± 1.6	−26.0 ± 0.9	−572 ± 1.8

**Table 2:** Mean number of HB between the fCNT carboxylic and carboxylate groups and the carbohydrate molecules determined using the following criterion: distance between acceptor and donor less than 3 Å, and angle acceptor-donor-H less than 20 deg.

	CNT-COO^⊖^	CNT-COOH	
	HB Acceptor	HB Acceptor	HB Donor
GlcA	2.51	0.47	0.02
GalNAc	8.71	1.03	0.02
GlcNAc	9.10	1.01	0.03
4HA	3.24	0.51	0.02
1HA	0.62	0.05	0.002
4Ch	2.61	0.56	0.03
1Ch	0.89	0.19	0.001

## Data Availability

The data that support the findings of this study are available from the authors.
